# *Dendrobium nobile* Polysaccharide Attenuates Blue Light-Induced Injury in Retinal Cells and In Vivo in *Drosophila*

**DOI:** 10.3390/antiox13050603

**Published:** 2024-05-14

**Authors:** Wei-Hsiang Hsu, Chanikan Sangkhathat, Mei-Kuang Lu, Wei-Yong Lin, Hsin-Ping Liu, Yun-Lian Lin

**Affiliations:** 1 Department of Chinese Pharmaceutical Sciences and Chinese Medicine Resources, China Medical University, Taichung 40402, Taiwan; d49818002@gm.ym.edu.tw (W.-H.H.); u112058101@cmu.edu.tw (C.S.); 2 National Research Institute of Chinese Medicine, Ministry of Health and Welfare, Taipei 11221, Taiwan; mklu@nricm.edu.tw; 3 Graduate Institute of Integrated Medicine, College of Chinese Medicine, China Medical University, Taichung 40402, Taiwan; linwy@mail.cmu.edu.tw; 4 Graduate Institute of Acupuncture Science, College of Chinese Medicine, China Medical University, Taichung 40402, Taiwan; 5 Department of Pharmacy, National Taiwan University, Taipei 10050, Taiwan

**Keywords:** *Dendrobium nobile*, polysaccharide, blue light, oxidative stress, opsin, phototransduction

## Abstract

Blue light is the higher-energy region of the visible spectrum. Excessive exposure to blue light is known to induce oxidative stress and is harmful to the eyes. The stems of *Dendrobium nobile* Lindl. (Orchidaceae), named Jinchaishihu, have long been used in traditional Chinese medicine (TCM) for nourishing yin, clearing heat, and brightening the eyes. The polysaccharide is one of the major components in *D. nobile*. However, the effect on ocular cells remains unclear. This study aimed to investigate whether the polysaccharide from *D. nobile* can protect the eyes from blue light-induced injury. A crude (DN-P) and a partially purified polysaccharide (DN-PP) from *D. nobile* were evaluated for their protective effects on blue light-induced damage in ARPE-19 and 661W cells. The in vivo study investigated the electroretinographic response and the expression of phototransduction-related genes in the retinas of a *Drosophila* model. The results showed that DN-P and DN-PP could improve blue light-induced damage in ARPE-19 and 661W cells, including cell viability, antioxidant activity, reactive oxygen species (ROS)/superoxide production, and reverse opsin 3 protein expression in a concentration-dependent manner. The in vivo study indicated that DN-P could alleviate eye damage and reverse the expression of phototransduction-related genes, including *ninaE*, *norpA, Gαq*, *Gβ76C*, *Gγ30A*, *TRP*, and *TRPL*, in a dose-dependent manner in blue light-exposed *Drosophila*. In conclusion, this is the first report demonstrating that *D. nobile* polysaccharide pretreatment can protect retinal cells and retinal photoreceptors from blue light-induced damage. These results provide supporting evidence for the beneficial potential of *D. nobile* in preventing blue light-induced eye damage and improving eyesight.

## 1. Introduction

Blue light is a higher-energy region of the visible spectrum. Blue light overexposure is widely known to induce oxidative stress and inflammatory response leading to mitochondrial dysfunction and DNA damage [1,2], which is harmful to the retina and ocular surface [3] and increases eye diseases such as dry eye, cataracts, and age-related macular degeneration (AMD) [4]. Various methods have been recommended to protect the eyes against blue light damage, but they cannot completely cure patients [3]. Chinese herbal medicine, in the context of treating retinal damage and improving eyesight, has paid much attention to these unmet needs.

The retina performs a crucial role in the process of visual transduction. Retinal photoreceptors (rod or cone cells) and retinal pigment epithelial (RPE) cells are involved in vision formation. The photoreceptors lie in the outer layer of the retina near the RPE and choroid [5,6]. The RPE serves multiple functions such as creating a blood–retinal barrier, transporting nutrients, providing protection against light and free radicals’ attacks, etc. [7]. Rhodopsin is a photosensitive G-protein-coupled receptor (GPCR) found in rod cells for detecting light/dark contrast, and mutations in the rhodopsin gene contribute to various retinal degenerative diseases such as retinitis pigmentosa [8]. Cone opsins are also photosensitive receptors in cone cells and detect color, being essential in retina vision formation [3,9]. Photoreceptor cells subjected to blue light can experience severe damage [10,11]. Blue light inhibits lysosomal autophagy via oxidative stress damage in the retina [12]. Other studies have also shown that photochemical injury involves oxidation, and photonic damage leads to the apoptotic death of retinal cells [13]. Furthermore, the development of AMD may be influenced by blue light [14].

The pseudobulb of *Dendrobium nobile* Lindl. (Orchidaceae), named Jinchaishihu, is one of the main sources of Dendrobii Caulis and is recorded in both “Shen Nong’s Herbal Classic” as well as the China Pharmacopoeia of the People’s Republic of China (2020) [15]. It is a homology of medicine and food. The fresh or dried stems are used in traditional Chinese medicine (TCM) for nourishing yin, clearing heat, and brightening the eyes [16,17,18]. Various chemical constituents such as alkaloids [19,20,21,22,23], sesquiterpenes [24,25,26], bibenzyls [27], phenanthrenes [19], and polysaccharides [18,22,23,28,29,30,31,32] have been identified as their active components, and their activity mechanisms have also been elucidated. Among them, polysaccharides, constituting one of the main active ingredients, have been reported for immunomodulation [29,33], antioxidative activity [30,31], anti-inflammatory activity [28], alleviating gastric ulcer [17,34], and having hypoglycemic [32], neuroprotective [35], and antitumor [36,37] effects.

*Drosophila* has a short life cycle, small body size, high reproductive capacity, and simple genome, which makes fruit flies excellent model organisms for various studies including in genetics, development, behavior, neurobiology, and investigations of human diseases [38]. Here, we used *Drosophila* as an in vivo organism to study the neuroprotection of polysaccharides extracted from *D. nobile* against blue light exposure. Similar to the vertebrates, the photoreceptors in the *Drosophila* retina absorb photos and perform visual transduction [39,40]. *Electroretinograms* (ERGs) can be used to measure electrical activity in response to light stimulation and to study the functionality of fly retinal systems. Within the ERG signal pattern, the receptor potential amplitude (RPA) represents the voltage difference of the photoreceptors’ depolarization during light stimulation. The on- and off-transient spikes, which occur at the beginning and the end of a flash of light, respectively, indicate the transmission of a signal from photoreceptors to their postsynaptic partners in the lamina [41,42].

Several studies have indicated that blue light harms retinal cells both in vitro and in vivo [11,43,44]. Polysaccharides constitute one of the major constituents in *D. nobile*, and have garnered considerable attention due to their various biological activities, but their effect on ocular cells remains unclear. This study aimed to investigate whether polysaccharide from *D. nobile* can protect eyes against blue-light-exposure harm.

## 2. Materials and Methods

### 2.1. Reagents

Fetal bovine serum (FBS), Dulbecco’s modified Eagle’s medium (DMEM), and DMEM Ham’s F12 medium (DMEM-F12) were purchased from Gibco (Billings, MT, USA). Unless otherwise stated, all chemicals were obtained from Sigma-Aldrich/Merck (St. Louis, MO, USA).

### 2.2. Herbal Materials

The dried stems, aged 1 year, of *D. nobile* were provided by Chih-Hsin Yeh, Ph.D., an expert in Taoyuan District Agricultural Research and Extension Station (TDARES), Taoyuan, Taiwan, where a voucher specimen was deposited at the National Museum of Natural Science of Taiwan (TNM, No. Yung-I Lee, 201602).

### 2.3. Preparation of Crude Polysaccharide

Dried ground stems of *D. nobile* were extracted with 80 °C hot water at a ratio of 1:100 (*w*/*w*) for 6 h twice and cooled. Then, the crude polysaccharide from *D. nobile* was prepared by adding 95% ethanol (1:4) and precipitating at 4 °C overnight. The precipitated polysaccharides were collected via centrifugation at 4000 rpm for 30 min. The precipitate was resuspended in Milli-Q water and kept at −20 °C overnight before lyophilization to yield the crude polysaccharides (DN-P).

### 2.4. Determination of Homogeneity of DN-P via Size-Exclusion Chromatography

The homogeneity of DN-P was determined via size-exclusion chromatography (SEC). Briefly, DN-P was dissolved in Milli-Q water to yield a concentration of 1 mg/mL before injection into the SEC columns (G4000PWXL 7.8 × 300 mm and G3000PWXL 7.8 × 300 mm) and eluted with Milli-Q water. The flow rate was 0.5 mL/min, monitored by RI detector. The molecular weight of the polysaccharides was determined through comparison with the pullulan standard of molecular weights of 78.8 × 10^4^, 40.4 × 10^4^, 21.2 × 10^4^, 4.73 × 10^4^, 1.18 × 10^4^, and 0.18 × 10^3^ Da (Sodex P-82 series; Showa Denko America, New York, NY, USA). Data analysis was performed using TriSEC conventional GPC software (TriSEC2000 v.1) [45].

### 2.5. Purification of DN-P through Gel Filtration Column Chromatography

The DN-P was reconstituted in a buffer solution comprising 10 mM NaH_2_PO_4_ and 150 mM NaCl pH 6.8. The sample was introduced to a Fractogel (BioSEC, Merck) column (103 × 1.5 cm) and run at a flow rate of (8 s/drops). A total of 100 drops per tube were collected (~2.8 mL/tube). Subsequently, phenol-sulfuric acid was employed to determine the carbohydrate-containing fractions through colorimetric analysis at 488 nm absorbance [45]. The molecular weight of each fraction was estimated by using authentic standards (dextran series, Sigma-Aldrich) with molecular weights of 670.0, 69.8, 40.0, 10.5, and 0.18 kDa. A regression equation was established by correlating log [Mw] (denoted *Y*) with the fraction number (denoted *X*) as follows: *Y* = 9.5145 − 0.1370*X* (*R*^2^ = 0.9972). Consequently, purified fractions were collected and dialyzed before freeze-drying to yield DN-PP.

### 2.6. Determination of the Monosaccharide Composition of Purified Polysaccharides

Acid hydrolysis of purified polysaccharide was performed using 1.95 M trifluoroacetic acid at 80 °C overnight. The mixture was cooled, evaporated, and resuspended in Milli-Q water. The acid hydrolysates were then separated via a high-performance anion exchange column chromatography (HPAEC) system (Dionex BioLC, Sunnyvale, CA, USA) with an anion-exchange column (Carbopac PA-10, 4.6 × 250 mm) and an amperometry detector (PAD-II). Monosaccharides were analyzed using an isocratic 18 mM NaOH elution at room temperature and identified and quantified through comparison with standards and interpolation from a standard curve. Data collection and analysis were performed utilizing a PRIME DAK system [45].

### 2.7. Cell Culture

The human retinal pigment epithelial cell line (ARPE-19) was purchased from Bioresource Collection and Research Center, Taiwan. Mouse photoreceptor-derived 661W cells were obtained from Professor Chang-Hao Yang, Department of Ophthalmology, College of Medicine, National Taiwan University, Taipei, Taiwan. ARPE-19 and 661W cells were cultured in DMEM/F12 medium and DMEM low glucose, respectively. The media were supplemented with 10% fetal bovine serum (FBS), 100 U/mL penicillin, and 100 mg/mL streptomycin. All cells were maintained in 100 mm culture dishes and incubated in a humidified atmosphere of 95% air and 5% CO_2_ at 37 °C.

### 2.8. Time Course Study of Blue Light Exposure in ARPE-19 and 661W Cells

ARPE-19 (5 × 10^3^ cells/well) and 661W (1.5 × 10^4^ cells/well) cells in 96-well plate were incubated in a normal medium for attachment. Then, the medium was replaced with DMEM/F12 (2% FBS) and DMEM low glucose (1% FBS). Cells were divided into two groups—(1) control: cells were cultured in a humidified atmosphere of 95% air and 5% CO_2_ at 37 °C and covered by aluminum foil; (2) blue light exposure: cells illuminated with the blue light source at a distance to keep the light intensity at 2500 lux [46] for 3 h, 6 h, and 16 h.

### 2.9. DN-P and DN-PP Protective Assay

ARPE-19 (5 × 10^3^ cells/well) and 661W (1.5 × 10^4^ cells/well) cells were seeded in 96-well plates. After overnight starvation, the cells were treated with (1) control (without any additional treatment); (2) blue light exposure only; (3) DN-P pretreatment (pretreated with 50, 100, and 200 μg/mL of DN-P); (4) DN-PP (mixture of F1 + F2 + F3) pretreatment (pretreated with 25, 50, and 100 μg/mL of DN-PP); and (5) α-lipoic acid (ALA) group (pretreated with 10, 25 and 50 μM of ALA), a positive control in in vitro and in vivo *Drosophila* experiments [43,47]. For cell protective assay, DN-P, DN-PP, and ALA were pretreated for 1 h before blue light exposure for 6 h.

### 2.10. Cell Viability Assay

The cell viability was detected using a Cell Counting Kit-8 (CCK-8) kit (Elabscience, Houston, TX, USA). After treatments, we added 10 μL of CCK-8 to each well, then incubated for 1 h before measurements. The absorbance was read at 450 nm using a microplate reader.

### 2.11. Measurements of ROS and Superoxide Generations, Antioxidant Activity, and Inflammatory Cytokines

Total ROS and superoxide production in ARPE-19 and 661W cells were detected using ROS-ID total ROS/superoxide detection kit (Enzo Life Science, Plymouth Meeting, PA, USA) and measured according to the manufacturer’s instructions. Fluorescence was read at excitation/emission: 488/520 nm (ROS detection) and 550/610 nm (superoxide detection). The experiments were repeated three times at least.

For antioxidant analysis, cells were harvested and homogenized using lysis buffer quickly, then centrifuged at 4 °C for 10 min. The activities of superoxide dismutase 1 (SOD 1) (Abcam, Cambridge, UK) and catalase (CAT) (Cayman, Ann Arbor, MI, USA) were determined using assay kits.

For measuring cytokine secretion, the culture supernatant was collected after 6 h of blue light exposure. The cytokines in supernatant were analyzed using commercially available ELISA kits (Invitrogen, Carlsbad, CA, USA). The total amount of IL-1β and IL-6 in the cell medium was normalized to total amount of protein in the viable cell pellets. The samples were analyzed in duplicate and repeated at least three times.

### 2.12. Western Blotting Analysis

Protein concentration was determined using a protein assay kit (Pierce, Rockford, IL, USA). Equal amounts of protein were separated on SDS-PAGE gel and transferred to polyvinylidene fluoride (PVDF) membranes. The membranes were blocked with 5% nonfat milk, then incubated with the primary antibodies (opsin 3 and tubulin) (Merck, Darmstadt, Germany) at 4 °C for overnight, followed by incubation with the secondary antibody for 1 h at room temperature. Protein expression was visualized by using a Western blot ECL substrate (Merck Millipore, NJ, USA). The optical density of the immunoreactive bands was visualized with Fujifilm LAS4000 luminescent image analysis system and quantified by using Multi-Gauge v3.0 software (Fujifilm, Tokyo, Japan).

### 2.13. Drosophila Model and Eye-Protective Experiments

#### 2.13.1. Fly Strain and Maintenance

*Drosophila w^1118^* strain was used and obtained from Bloomington *Drosophila* Stock Center (BDSC), Bloomington, USA. Flies were fed with cornmeal–sucrose–yeast culture medium under 12 h/12 h light/dark cycle of 25 °C and 60% humidity and transferred to fresh medium every 3–4 days. For evaluating the impact of blue light and the protective effect of DN-P on fly eyes, male flies were collected within 1 day after eclosion and without or with DN-P (2.5 and 12.5 mg/mL) and ALA (2, 4, and 8 mM, as a positive control) for 10 days, following our previous preparation [48].

#### 2.13.2. ERG Assay

The setting up of ERG experiment was performed according to our previous study [49]. The fly eye was exposed to a 6.5–7.0 klux white-light LED, which is programmed to engage in a repetitive on–off cycle of 2–6 s. The ERG signal of each fruit fly was recorded for six cycles using Axon Instruments GeneClamp 500 Voltage Patch Clamp Amplifier (Molecular Devices, San Jose, CA, USA) and analyzed using Axon Instruments AxoScope 10.2 software (Molecular Devices, San Jose, CA, USA). For evaluating the impact of blue light, the adult male flies (1 day after eclosion) were sustained without (as the control group) or with DN-P or ALA for 10 days. Then, the eyes of flies were exposed to blue light (λ = 450 nm) with an intensity of 140 klux from a light source (JLD45160ZA-N1Y, Nichia, Anan-Shi, Japan). The sensitivity to light was calculated using the values of ΔV, RPA, on- and off-transient amplitude after blue light exposure and normalized to compare the values of ΔV, RPA, and on- and off-transient amplitude before blue light exposure. At least 14 replicates for each group were observed.

### 2.14. Quantitative Polymerase Chain Reaction (qPCR)

For qPCR analysis, fly retinas were dissected and stored at −80 °C. Total RNA was extracted using the sample lysis buffer (150 mM NaCl, 10 mM Tris pH8, 5 mM DTT, 40U RNase OUT). cDNA was synthesized using a High-Capacity cDNA Reverse Transcription Kit (Applied Biosystems, Waltham, MA, USA) following the manufacturer’s protocol. qPCR reaction was prepared using LightCycler 480 SYBR Green I Master mix (Roche, Basel, Switzerland) and assayed with ViiA 7 Real-Time PCR System (Thermo Fisher Scientific, Waltham, MA, USA). The primers sequences for the target genes were as follows: *Rpl32*_F (5′-CGG ATC GAT ATG CTA AGC TGT-3′), *Rpl32*_R (5′-CGA CGC ACT CTG TTG TCG-3′), *ninaE*_F (5′-GAG GGC CTT ACA CCA CTG AA-3′); *ninaE*_R (5′-CGA TAT TTC GGA TGG CTG AT-3′), *norpA*_F (5′-TCT TTG AGC CTG TCA CGT TG-3′), *norpA*_R (5′-TCT TTG CTC TTG CCC TTG AT-3′), *Gαq*_F (5′-AGG ACA AGC GTG GGT ACA TC-3′), *Gαq*_R (5′-CTC GAA CGT GGT AAC GGT CT-3′), *Gβ76C*_F (5′-TAT CGC TGG CTT ATC GCT TT-3′), *Gβ76C*_R (5′-TCG TGA CCG AAG AAC ATC TG-3′), *Gγ30A*_F (5′-CGC TGG CCG TTA TCT AAA TC-3′), *Gγ30A*_R (5′-GGC CCA TGG ATT GTT CTT C-3′), *trp*_F (5′-GAT TAC GGC ATT ACC GAG GA-3′), *trp*_R (5′-CAA CTC CCT GCG ACT TCT TC-3′), *trpl*_F (5′-GAA CAG CGG AAT GGA TGT TT-3′), and *trpl*_R (5′-TGG ACT CCA CCT TGA TCT CC-3′). *Rpl32* is a housekeeping gene. The results were quantified using the comparative 2^−ΔΔCt^ method, and the data of the control group without exposure to blue light were treated as normalized data. Five replicates for each group were observed.

### 2.15. Statistical Analysis

All data represent means ± standard errors of the mean (SEMs). The significance of the difference between the control and experimental groups was evaluated with Student’s *t*-test, and multiple groups were assessed with Turkey’s one-way ANOVA using GraphPad Prism 8.0.2. All calculated *p*-values were two-tailed. #, * *p <* 0.05; ##, ** *p <* 0.01; and ###, *** *p <* 0.001 were defined as denoting statistical significance.

## 3. Results

### 3.1. Characterization of Crude (DN-P) and Purified (DN-PP) Polysaccharides

**Figure 1:**
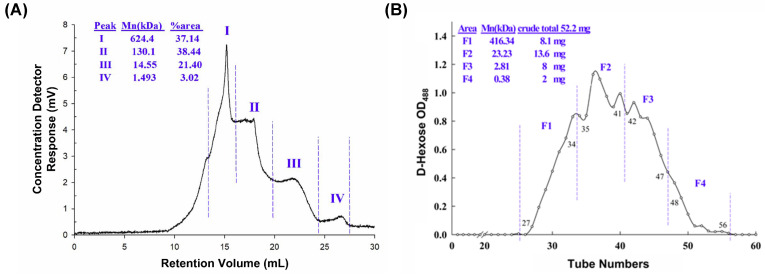
Chemical characterization of (**A**) crude (DN-P) and (**B**) purified (DN-PP) polysaccharides from *D. nobile*. (**A**) The average molecular weight distribution of DN-P was detected using an RI detector and comparison with pullulan standard and analyzed using TriSEC conventional GPC software. (**B**) DN-P was purified through gel filtration to yield four major fractions. The average molecular weights of fractions F1 (27–34), F2 (35–41), F3 (42–47), and F4 (48–56) by using dextran authentic standards were 416.34, 23.23, 2.81 and 0.38 kDa, respectively. The yields of the fractions (from 52.2 mg of DN-P) were 8.1, 13.6, 8, and 2 mg, respectively. F1~F3 mixed together to be DN-PP.

The yield of DN-P is 10.84% of the dried stem of *D. nobile*. DN-P is a heterogeneous polysaccharide containing four clusters. The average molecular weights (Mn) of four polysaccharides were 624.4 (37.14%), 130.1 (38.44%), 14.55 (21.40%), and 1.493 (3.02%) kDa (Figure 1A). DN-P was further purified through gel filtration, four fractions were collected, and their average molecular weights (Mn) were 416.34, 23.23, 2.81, and 0.38 kDa (Figure 1B). The yield of purified polysaccharides (F1, F2, and F3) was about 57% of DN-P. Fraction four, possessing a molecular weight of 0.38 kDa, exhibited a lower propensity to comprise polysaccharides. Consequently, only the crude polysaccharide (DN-P) and three fractions (F1, F2, and F3) identified the monosaccharide compositions (Table 1).

The analysis of monosaccharide compositions in DN-P and three fractions in DN-PP showed that the major monosaccharide constituents were mannitol, glucose, and mannose. Mannose was the highest component, and little fructose was exclusively detected in F1 and F2 with 11.44 and 5.72 μmol/g of polysaccharide, respectively (Table 1). Because F1, F2, and F3 were not well separated and the yield of each fraction was limited. F1 (8.1 mg), F2 (13.6 mg), and F3 (8 mg) were then mixed together as DN-PP for subsequent in vitro studies.

### 3.2. Blue Light-Induced ARPE-19 and 661W Cell Damage

**Figure 2:**
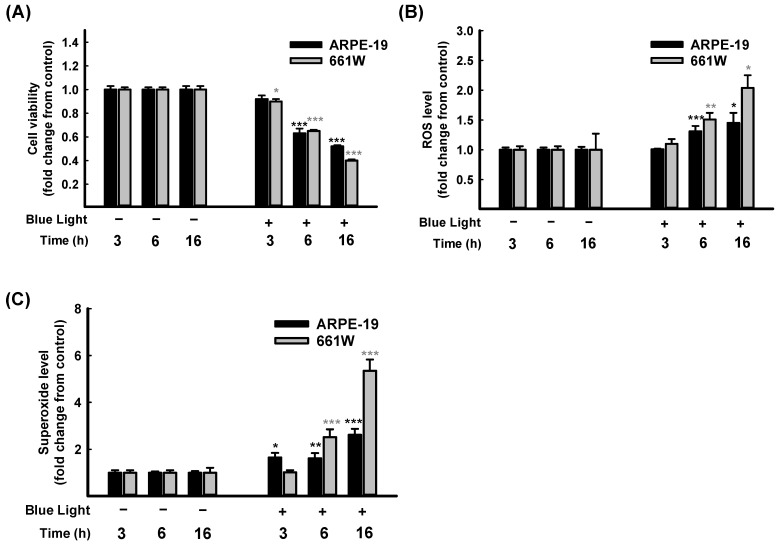
Time course study on blue light-induced ARPE-19 and 661W cell damage. Blue light illuminated the cells for 3 h, 6 h, and 16 h; cell viability of ARPE-19 and 661W cells were evaluated through CCK-8 assay (**A**) Levels of ROS (**B**) and superoxide (**C**) in ARPE-19 and 661W cells were determined using kits. Data are represented as means ± SEMs from three independent experiments. * *p* < 0.05, ** *p* < 0.01, and *** *p* < 0.001, compared with control group.

As far as we know, blue light exposure increases ocular pathologies. The mechanisms involved in blue light injury are mainly associated with oxidative stress and cell apoptosis [1,2]. The retina performs a crucial role in the process of visual transduction. Retinal photoreceptors (rod or cone cells) and RPE cells are involved in vision formation [3]. Consequently, from a human retinal pigment epithelial cell line, ARPE-19, and a mouse-derived photoreceptor cell line, 661W, cells were selected for further in vitro investigation. First of all, a time course study of blue light exposure was performed. As Figure 2 shows, the cell viability decreased in both ARPE-19 and 661W cells compared to the control group. The cell viability reduction was in a time course-dependent manner and with a significance of *p* < 0.001 at 6 h and 16 h (Figure 2A).

Further, the intracellular ROS/superoxide releases in ARPE-19 and 661W cells were determined after blue light illumination. As expected, both intracellular ROS (Figure 2B) and superoxide (Figure 2C) levels increased in a time course-dependent manner. Overall, 661W cells are more sensitive than ARPE-19 cells. These results confirmed that exposure to blue light-induced oxidative stress in retinal cells led to a decrease in cell viability in both ARPE-19 and 661W cells in a time-dependent manner. The cell viability decreased to lower than 50% after 16 h of exposure (Figure 2A). Therefore, exposure for 6 h was selected for the following protective experiments.

### 3.3. DN-P and DN-PP on Blue Light-Induced ARPE-19 and 661W Cell Damage

**Figure 3:**
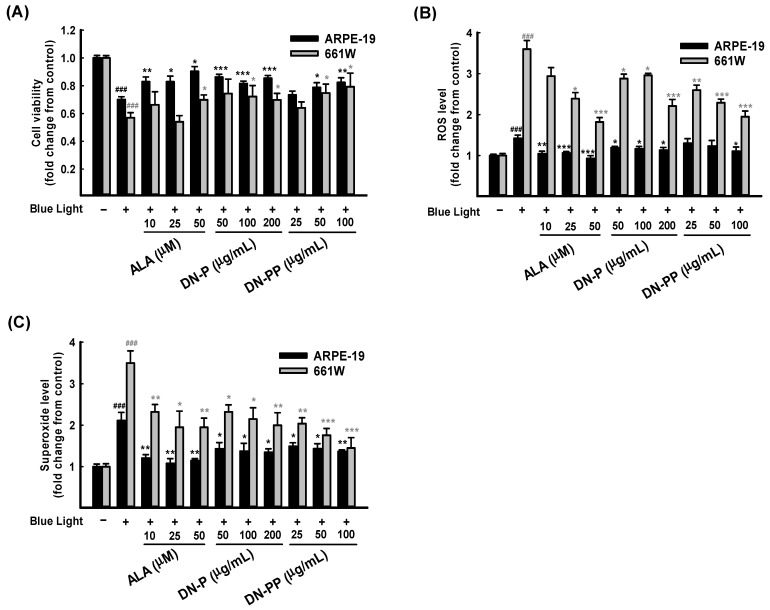
DN-P and DN-PP on blue light-induced injury in ARPE-19 and 661W cells. ARPE-19 and 661W cells were pretreated with different concentrations of DN-P and DN-PP, then treated without or with blue light exposure for 6 h of incubation. (**A**) Cell viability values in ARPE-19 and 661W cells were measured via CCK-8 assay. Levels of ROS (**B**) and superoxide (**C**) in ARPE-19 and 661W cells were determined using kits. ALA was a positive control. Data are represented as means ± SEMs from three independent experiments. ### *p* < 0.001, compared with the control group. * *p* < 0.05, ** *p* < 0.01, and *** *p* < 0.001 compared with blue light treatment only.

After 6 h of blue light exposure, the cell viability of ARPE-19 cells decreased to 0.70 ± 0.02 compared to the control group. However, the viability of the DN-P pretreatments with 50, 100, and 200 μg/mL were 0.85 ± 0.02, 0.81 ± 0.01 and 0.86 ± 0.01, respectively. DN-PP pretreatments at 25, 50 and 100 μg/mL observed the cell viability values of 0.73 ± 0.02, 0.78 ± 0.03 and 0.82 ± 0.03, respectively. Upon pretreatment with ALA (as positive control) at 10 and 25 μg/mL, the cell viability values were 0.82 ± 0.03 and 0.82 ± 0.04, respectively. The cell viability in all pretreatment groups mentioned above was higher than that of the blue light treated-only group. All of the presented values were statistically significant except DN-PP at 25 μg/mL, and similar results were obtained for all pretreatments in 661W cells (Figure 3A). As the above results showed, DN-P and DN-PP could effectively protect ARPE-19 and 661W cells from blue light-induced cell death.

Blue light injury increases oxidative stress, which is involved in cell death [13,14]. Therefore, we evaluated the intracellular release of ROS and superoxide in ARPE-19 and 661W cells in the same way as the above treatments related to cell viability. As Figure 3B shows, exposure to blue light significantly increased ROS and superoxide levels in 661W cells. However, the increased level in ARPE-19 cells was relatively low. DN-P, DN-PP, and ALA pretreatments with various concentrations decreased ROS levels in a concentration-dependent manner in 661W cells but not in ARPE-19 cells.

Regarding superoxide release, pretreatment groups exhibited various protective effects in both ARPE-19 and 661W cells (Figure 3C). A comparison of DN-P and DN-PP pretreatments and blue light treated-only group revealed significant decreases in superoxide levels and a similar trend to ROS inhibition in both ARPE-19 and 661W cells. These findings indicate that the pretreatment with DN-P and DN-PP can effectively abrogate intracellular ROS and superoxide levels in ARPE-19 and 661W cells.

**Figure 4:**
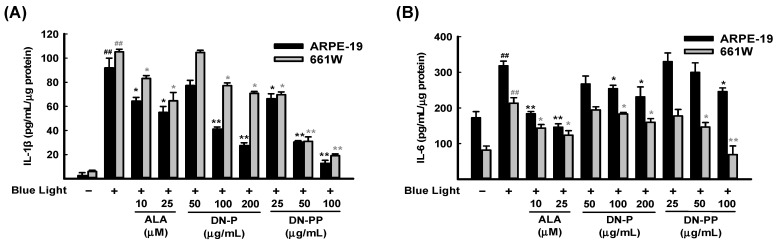
DN-P and DN-PP on pro-inflammatory cytokines in blue light-exposed ARPE-19 and 661W cells. Cells were pretreated with DN-P and DN-PP at the indicated concentrations for 1 h and then incubated with blue light exposure for 6 h. The secretion levels of (**A**) IL-1β and (**B**) IL-6 were assessed using ELISA kit. ALA was a positive control. Data are expressed as means ± SEMs of three independent experiments. ## *p* < 0.01 vs control group; * *p* < 0.05 and ** *p* < 0.01 vs blue light exposure.

Narimatsu et al. (2013) reported that blue light exposure upregulates inflammatory cytokines in RPE-choroid in response to light-induced ROS [50]. Therefore, we measured the levels of inflammatory cytokines IL-1β and IL-6 in ARPE-19 and 661W cell medium after 6 h of blue light exposure. As expected, IL-1β (Figure 4A) and IL-6 (Figure 4B) were greatly increased in both cells after blue light exposure and were inhibited by DN-P and DN-PP pretreatments in a concentration-dependent manner.

### 3.4. DN-P and DN-PP on Blue Light-Induced Antioxidant Activity in ARPE-19 and 661W Cells

**Figure 5:**
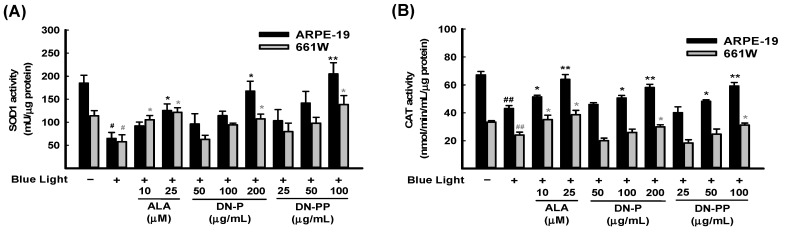
DN-P and DN-PP on antioxidant activity in blue light-induced ARPE-19 and 661W cells. ARPE-19 and 661W cells were pretreated with different concentrations of DN-P and DN-PP for 1 h, then treated without or with blue light exposure for 6 h. The antioxidant activities of SOD1 (**A**) and catalase (CAT) (**B**) in ARPE-19 and 661W cells were measured using kits. ALA was a positive control. Data are represented as means ± SEMs from three independent experiments. # *p* < 0.05 and ## *p* < 0.01 compared with the control group. * *p* < 0.05 and ** *p* < 0.01 compared with blue light treatment only.

In addition to ROS/superoxide generation in ARPE-19 and 661W cells, the protective effect of DN-P and DN-PP on the activities of antioxidant enzymes, including SOD1 and CAT, was also determined. The activities of SOD1 and CAT in the blue light group were significantly lower than in the control group (Figure 5A,B). Pretreatment with DN-P and DN-PP concentration-dependently mitigated blue light-reduced SOD1 and CAT levels (Figure 5A,B). Thus, pretreatment with DN-P and DN-PP increased antioxidant activity to attenuate blue light-induced ROS/superoxide generation.

### 3.5. DN-P and DN-PP on Blue Light-Induced Opsin Expression in 661W Cells

**Figure 6:**
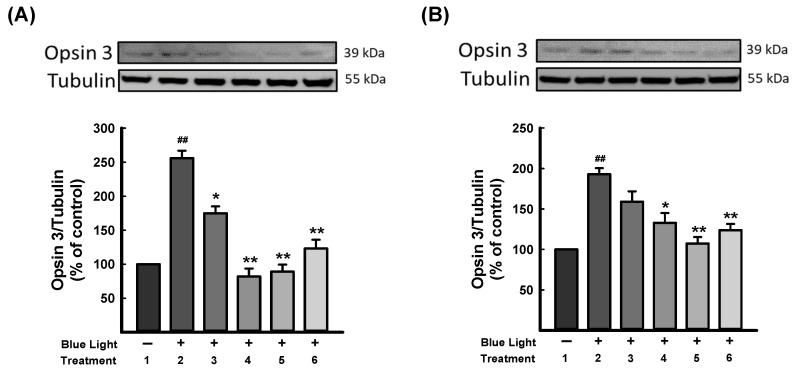
DN-P and DN-PP on opsin 3 expression in blue light-induced injury in 661W and ARPE-19 cells. (**A**) 661W and (**B**) ARPE-19 cells illuminated without treatment (1) and with blue light (2) illumination only and with DN-P (3, 200 and 4, 400 μg/mL), DN-PP (5, 100 μg/mL), and ALA (6, 10 μM) for 1 h before blue light exposure for 6 h. Then, cells were harvested for Western blotting analysis with the indicated antibodies. The expression of tubulin was an internal control. ALA was a positive control. Data are means ± SEMs from three independent experiments. ## *p* < 0.01 vs control; * *p* < 0.05 and ** *p* < 0.01 vs blue light-illuminated cells.

Opsins are G-protein-coupled proteins and are prominently found in photoreceptor cells of the retina, which mediate light sensitivity [51]. Ratnayake et al. (2018) reported that blue light could activate opsins and excite retinal to intercept crucial signaling molecules and change the cellular fate in the retina [52]. As Figure 6 shows, blue light exposure was significantly increased in opsin 3 expression. DN-P 400 μg/mL and DN-PP 100 μg/mL reversed opsin 3 expression to a similar level as a control group in 661W cells, as did DN-PP 100 μg/mL in ARPE-19 cells.

### 3.6. DN-P Pretreatment on Blue Light-Induced Retinal Damage in Fly Eyes, Evaluated via ERG Analysis

**Figure 7:**
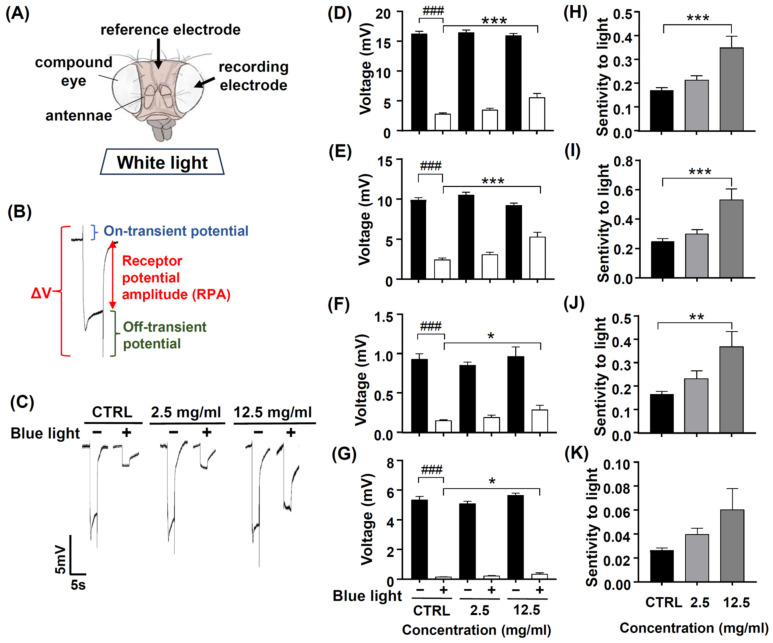
The protective effect of DN-P on blue light-induced retinal damage in fly eyes. Flies were pretreated with 0, 2.5, and 12.5 mg/mL DN-P for 10 days. (**A**) The position of reference and recording electrodes. (**B**) ERG signal represents fly visual transduction with ΔV, RPA, and on-transient and off-transient amplitudes. (**C**) The representative ERG signals of the control and treated groups. The characteristics of the ERG were recorded by the amplitudes of (**D**) ΔV and (**E**) RPA and (**F**) on-transient (**G**) and off-transient amplitudes. The sensitivity to light of (**H**) ΔV, (**I**) RPA, and (**J**) on-transient and (**K**) off-transient amplitudes. Data are means ± SEMs. n = 14–15. ### *p* < 0.001 with vs. without blue light exposure. * *p* < 0.05, ** *p* < 0.01, and *** *p* < 0.001 compared to the control group.

Photoreceptors respond to light and induce phototransduction by converting light signals into electric currents, and thus, an extracellular ERG can record a periodic electric signal (Figure 7A). The typical ERG waveform of a control fly can be divided into four components (Figure 7B). The receptor potential amplitude (RPA) reflects the photoreceptors’ depolarization during light stimulation. The on- and off-transient spikes, which occur at the beginning and end of a flash of light, respectively, indicate the successful transmission of a signal from photoreceptors to their postsynaptic partners in the lamina. The quantity ΔV represents the amplitude between on- and off-transient spikes.

Chen et al. (2017) showed that the antioxidant ALA has protective effects on blue light-induced retinal degeneration; therefore, we used ALA as a positive control [43]. After pretreating ALA 2, 4, and 8 mM for 10 days, the ERG signals of ΔV, RPA, and on- and off-transient amplitudes were similar to those of the control groups (Supplementary Figure S1A–D). After exposure to blue light, the ERG electric potentials significantly reduced, as indicated by the smaller ΔV, RPA, and on- and off-transient amplitudes (Supplementary Figure S1A–D, ### *p* < 0.001), and pretreated ALA 8 mM, ΔV, and RPA amplitudes were significantly larger compared to the no-ALA control group (Supplementary Figure S1A–D, * *p* < 0.05 and *** *p* < 0.001), suggesting that ALA appeared to have neuroprotective effects against blue light injury. We also analyzed the sensitivity to light of four components of ERG (ΔV, RPA, and on- and off-transient amplitudes); the values of ΔV, RPA, and on- and off-transient amplitude after blue light exposure were normalized to compare the values of ΔV, RPA, and on- and off-transient amplitude before blue light exposure. Our data found that the majorly affected ERG components are ΔV and RPA (Supplementary Figure S1E–H, ** *p* < 0.01 and *** *p* < 0.001, respectively). The ERG electrical traces of the control and ALA-treated groups are represented in Supplementary Figure S1I. These results suggest that ALA 8 mM has neuroprotective effects and attenuates blue light injury to the fly’s retina.

We tried to know whether exposure to blue light affected retinal phototransduction. After pretreating DN-P 2.5 and 12.5 mg/mL for 10 days, the ERG amplitudes of ΔV, RPA, on- and off-transient were similar to those of the control groups (Figure 7C–G). After exposure to blue light, ERG electric potentials were significantly reduced, as indicated by the smaller ΔV, RPA, and on- and off-transient amplitudes (Figure 7D–G, ### *p* < 0.001). Pretreating 12.5 mg/mL for 10 days, DN-P appeared to have neuroprotective effects against blue light injury. The four components (ΔV, RPA, and on-and off-transient amplitudes) of ERG were significantly larger in the DN-P 12.5 mg/mL group compared to the no-DN-P control group (Figure 7D–G, * *p* < 0.05 and *** *p* < 0.001). In addition, we analyzed the sensitivity to light of four components of ERG (ΔV, RPA, and on-and off-transient amplitudes) before and after blue light exposure. Our data indicated that majorly affected ERG components are ΔV, RPA, and on-transient spike (Figure 7H–K, ** *p* < 0.01 and *** *p* < 0.001), suggesting that the pretreatment of DN-P 12.5 mg/mL has neuroprotective effects against blue light-induced retinal damage in fly eyes.

### 3.7. DN-P on Phototransduction-Related Gene Expression in Drosophila Retina

**Figure 8:**
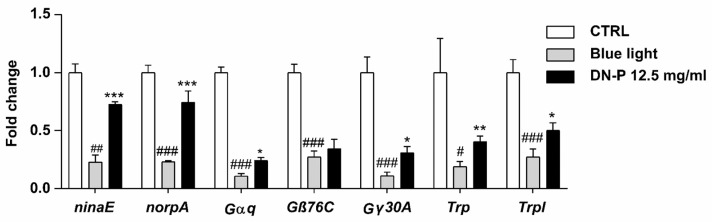
Effect of DN-P pretreatment on the gene expression levels of *Drosophila* retina. Data are means ± SEMs from five independent experiments. # *p* < 0.05, ## *p* < 0.01, and ### *p* < 0.001 compared to the control group. * *p* < 0.05, ** *p* < 0.01, and *** *p* < 0.001 compared to blue light treatment only.

In fly, light-induced phototransduction cascade activates rhodopsin (*ninaE*), heterotrimeric G-protein (*Gαq*, *Gβ76C*, and *Gγ30A*), and phospholipase C (*norpA*), then triggers open cation channels including transient receptor potential (*Trp*) and TRP-like protein (*Trpl*) channels [53]. Thus, we analyzed these phototransduction-related genes in the *Drosophila* retina using qPCR. Our data observed that blue light illumination significantly reduced the expression levels of *ninaE*, *norpA*, *Gαq*, *Gβ76C*, *Gγ30A*, *Trp*, and *Trpl* (Figure 8, # *p* < 0.05, ## *p* < 0.01, and ### *p* < 0.001). The pretreatment of 12.5 mg/mL DN-P exhibited a protective effect against the blue light injury to the retina, displaying rescued effects and increasing expressions of *ninaE*, *norpA*, *Gαq*, *Gγ30A*, *Trp*, and *Trpl* compared to the blue light-only group (Figure 8, * *p* < 0.05, ** *p* < 0.01, and *** *p* < 0.001). Similar results were also observed in the 8 mM ALA group as a positive control (Supplementary Figure S2, #, * *p* < 0.05, ** *p* < 0.01, and ###, *** *p* < 0.001). Our studies indicated that exposure to blue light reduced phototransduction as per functional ERG analysis and the expression of phototransduction-related genes. Pretreatments of DN-P and ALA significantly attenuate blue light injury, suggesting that DN-P and ALA have neuroprotective effects against blue light-induced eye damage and improve eyesight.

## 4. Discussion

Various polysaccharides from *D. nobile* have been reported and characterized, and these are composed of several different molecular weight ranges and monosaccharides [19,31,32,54]. Yang et al. (2010) reported that the polysaccharide content in *D. nobile* varies depending on different organs, habitat, and growth period and that it reaches the highest peak in the second year (2.83%), then decreases in the third year [55]. Yan et al. (2018) reported that the yield peak at 17.32% was much higher levels [56]. In this study, *D. nobile* was provided by TDARES in Taiwan. We found the yield peak of the polysaccharide content at one year (10.84%). Furthermore, Luo et al. (2010) identified polysaccharides from *D. nobile* with a molecular weight of about 87.6 kDa, and these are composed of rhamnose, arabinose, xylose, mannose, glucose, and galactose in a molar ratio of 1.00:2.80:2.20:30.76:117.96:31.76 with antioxidant activity. Further, four major polysaccharide fractions obtained through column chromatography showed that their average molecular weights (Mn) were 136, 27.7, 11.8, and 11.4 kDa [31]. Our study found that the molecular weight distribution and the monosaccharide composition of our *D. nobile* polysaccharides were different from those in previous reports. Due to different growth environments and preparation methods, the yield and the composition of polysaccharides may differ and have different biological activities. Glucomannan is one of the major polysaccharides in the cell wall of *Dendrobium* species including *D. nobile*, and consists of glucose and mannose linked by glycosidic linkages [57]. In this study, mannose, glucose, and mannitol account for the major composition of monosaccharides. This may suggest that these molecules contribute to a high degree of protection effect of DN-PP. A similar study of *Dendrobium* polysaccharides (DNLP) found that they consisted of rhamnose, arabinose, xylose, mannose, glucose, and galactose in a molar ratio of 1.00:1.65:2.58:1.08:1.83:1.15, exhibiting the protection effect of fibroblasts against UVA-induced photoaging [58]. This suggested that other molecules like arabinose and xylose may also play roles in the protection effect of UVA-damaged fibroblasts.

Blue light is a part of the higher energy of the visible light spectrum and is widely known to be harmful to the retina and ocular surface [12,59]. The mechanisms involved in blue light injury are mainly associated with oxidative stress, mitochondrial dysfunction, inflammatory response, etc. [1,2,60]. Otsu et al. (2020) discovered that 661W cell exposure to blue light promoted oxidative stress and increased lysosomal cell death [13]. We here confirmed that exposure to blue light-induced oxidative stress in retinal cells led to a cell viability decrease (Figure 2) and enhanced ROS/superoxide (Figure 3) and inflammatory cytokines (Figure 4) in both ARPE-19 and 661W cells in a time-dependent manner. DN-P and DN-PP pretreatments attenuated those damages (Figure 2, Figure 3 and Figure 4) and increased both antioxidant activity (Figure 5) as well as opsin 3 expression in both ARPE-19 and 661W cells (Figure 6). Overall, our results found that the photoreceptor 661W cells are more sensitive than ARPE-19 cells. These results are consistent with Narimatsu et al. (2015), who reported that blue light is more harmful to photoreceptor cells [61]. Overall the yield of DN-PP is about half that of DN-P (57%) and the activity of DN-PP is equivalent to half the concentration of DN-P (Figure 3, Figure 4, Figure 5 and Figure 6), with even higher activities in IL-1β and IL-6 of DN-PP (Figure 4).

Several previous studies indicated that blue light harmed retinal cells both in vitro and in vivo [10,11,43]. In vitro cell lines and in vivo *Drosophila* models are parts of two commonly used methods for studying the effects of natural or synthetic substances on antioxidant stress. While in vitro studies offer a controlled environment for investigating cellular mechanisms, in vivo models provide a more realistic representation of the whole organism. Despite the differences between these two approaches, there is growing evidence that results obtained from in vitro cell lines can be consistent with those from in vivo *Drosophila* models [43,62,63,64]. Our results showed that exposure to blue light caused injury not only to the ARPE-19 and 661W cells but also to *Drosophila*’s phototransduction (Figure 7 and Figure 8). Through ERG analysis, blue light exposure resulted in lower amplitudes of RPA, on- and off-transients and DN-P pretreatment protected fly retina from blue light injury. In *Drosophila*, the phototransduction process occurs in the photoreceptors of ommatidia. In the photoreceptors, there are cell bodies and rhabdomeres, the specialized visual organelles composed of thousands of microvilli and harboring molecules that are necessary for the transduction of a captured photon of light. Visual information is detected by the retina, and visual processing occurs in the optic lobes [65]. The rhodopsin, a GPCR, can be activated through light stimulation [66]. After photoisomerization, rhodopsin activates a Gαq protein, triggers signal transduction through phospholipase C, and then opens cation channels. ERG is an extracellular recording to measure the light response of photoreceptors and laminar neurons in the optic lobe. The magnitudes of the ERG amplitudes reflect the sensitivity to the light in the fly retina. According to Damulewicz et al. (2019), a higher expression of cation channels *Trp* and *Trpl* increased the retina’s sensitivity to light [67]. Our studies found that exposure to blue light reduced the gene expression of the phototransduction pathway, suggesting a functional decrease in light sensitivity. DN-P and ALA pretreatments significantly increased the phototransduction-related gene expression, and these results indicate that DN-P and ALA attenuate blue light injury and have neuroprotective effects on photoreceptors.

## 5. Conclusions

We here conducted in vitro and in vivo studies to investigate the effects of polysaccharides from *D. nobile* on the antioxidant properties and elucidate the potential underlying mechanisms. As we know, this was the first study to demonstrate that pretreated polysaccharides from *D. nobile* reserve a protective effect. These results provide supporting evidence for the beneficial potential of *D. nobile* in preventing blue light-induced eye damage and improving eyesight.

## Figures and Tables

**Table 1:** Monosaccharide compositions of crude polysaccharide (DN-P) and three fractionated polysaccharides.

	Monosaccharides (μmol/g Polysaccharide)			
	Crude	F1	F2	F3
**sorbitol**	33.76 ± 2.92	30.08 ± 0.01	27.52 ± 0.26	26.30 ± 0.01
**mannitol**	213.60 ± 2.09	155.05 ± 0.45	201.23 ± 1.52	178.29 ± 0.37
**arabinose**	12.27 ± 0.62	27.72 ± 0.13	24.55 ± 0.28	12.86 ± 0.10
**galactose**	21.09 ± 0.10	37.73 ± 0.06	34.32 ± 0.07	18.04 ± 0.01
**glucose**	738.08 ± 2.25	275.67 ± 0.36	578.02 ± 0.93	441.72 ± 0.60
**mannose**	748.15 ± 1.65	721.11 ± 1.49	819.35 ± 1.72	645.28 ± 1.40
**fructose**	-	11.44 ± 0.05	5.72 ± 0.05	-

## Data Availability

Data will be made available on request.

## Supplementary Materials

The following supporting information can be downloaded at: https://www.mdpi.com/article/10.3390/antiox13050603/s1, Figure S1: Effect of ALA on blue light-induced retinal damage in fly eyes.; Figure S2: Effect of ALA pretreatment on the gene expression levels of *Drosophila* retina.

## Abbreviations

**Full Name**	**Abbreviation**
Age-related macular degeneration	AMD
Average molecular weight	Mn
Catalase	CAT
*Dendrobium*	*D.*
Dulbecco’s modified Eagle’s medium	DMEM
Crude polysaccharide of *D. nobile*	DN-P
Purified polysaccharide of *D. nobile*	DN-PP
Electroretinogram	ERG
Fetal bovine serum	FBS
G-protein-coupled receptor	GPCR
neither inactivation nor afterpotential E	*ninaE*
Reactive oxygen species	ROS
Superoxide dismutase	SOD
Retinal pigment epithelial	RPE
Size-exclusion chromatography	SEC
Traditional Chinese medicine	TCM
High-performance anion exchange column chromatography	HPAEC
Transient receptor potential	*Trp*
Transient receptor potential-like protein	*Trpl*
