# Supplementary material for: Dendrobium nobile Polysaccharide Attenuates Blue Light-Induced Injury in Retinal Cells and In Vivo in Drosophila
Source: Antioxidants (Basel). 2024 May 14;13(5):603. doi: 10.3390/antiox13050603 (PMC11118839; doi:10.3390/antiox13050603)
Supplement: Supplementary file 1 [file antioxidants-13-00603-s001.zip › antioxidants-2977718-supplementary.pdf]

Supplementary Figures

Figure S1

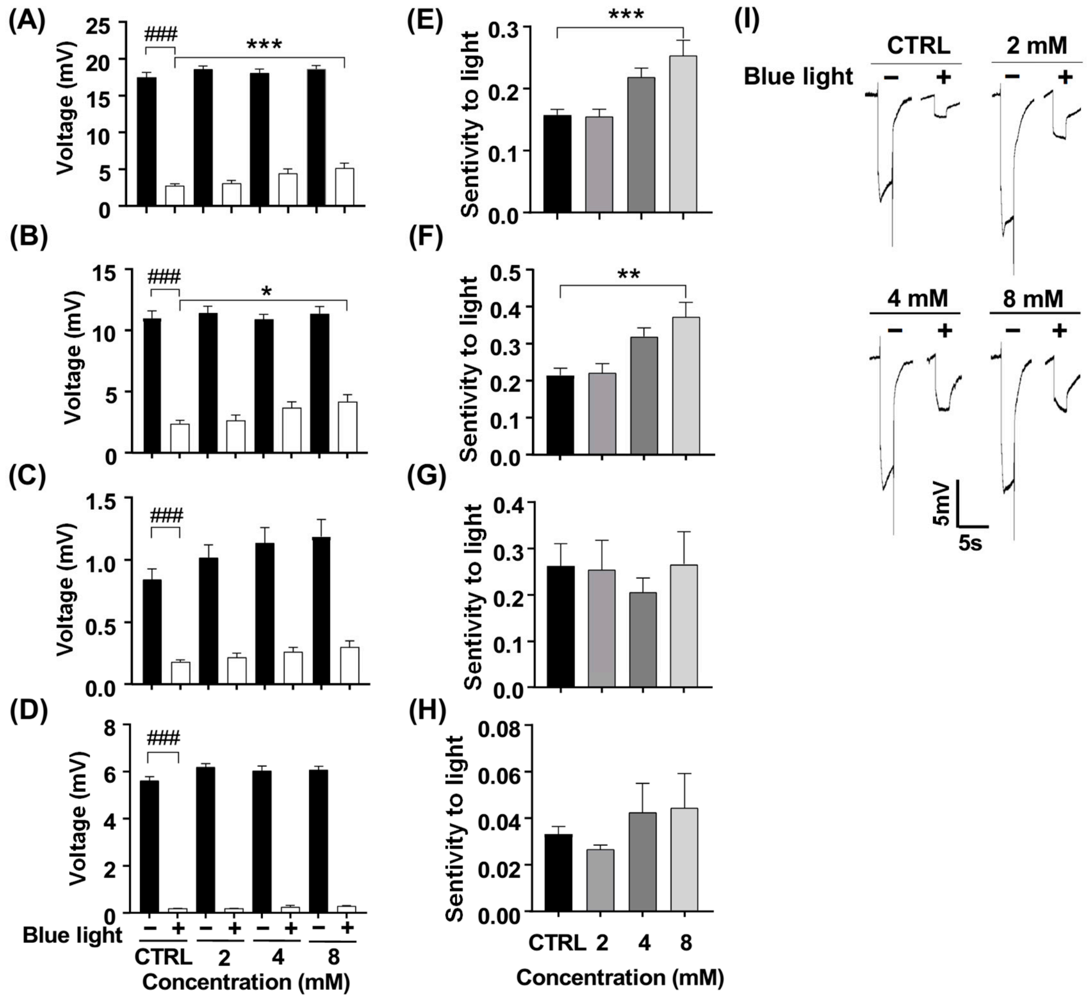

Figure S1. Effect of ALA on blue light-induced retinal damage in fly eyes. Male flies were pretreated with 0, 2, 4, and 8 mM ALA for 10 days. The characteristics of the ERG were recorded by the amplitudes of (A) ΔV, (B) RPA, (C) on-transient, and (D) off-transient. The sensitivity to light of (E) ΔV, (F) RPA, (G) on-transient, and (H) off-transient. (I) The representative ERG signals of the control and treated groups. Data are mean ± SEM. n = 14-15. ### $p < 0.001$ with vs. without blue light exposure. * $p < 0.05$, ** $p < 0.01$, and *** $p < 0.001$ compared to the control group.

Figure S2

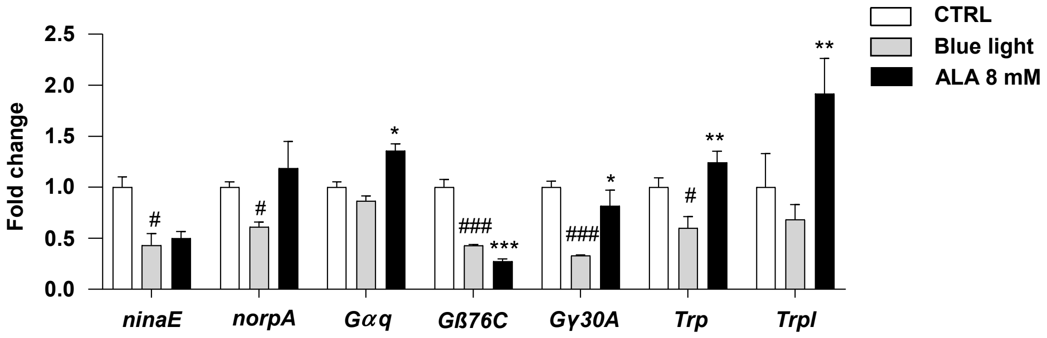

Figure S2. Effect of ALA pretreatment on the gene expression levels of *Drosophila* retina. ALA is a positive control. Data are mean ± SEM from five independent experiments. # $p < 0.05$, ### $p < 0.001$ compared to the control group. *$p < 0.05$, ** $p < 0.01$, *** $p < 0.001$ compared to blue light treatment only.
